# Supplementary material for: Acquisition of Colistin Resistance Links Cell Membrane Thickness Alteration with a Point Mutation in the lpxD Gene in Acinetobacter baumannii
Source: Antibiotics (Basel). 2020 Apr 6;9(4):164. doi: 10.3390/antibiotics9040164 (PMC7235794; doi:10.3390/antibiotics9040164)
Supplement: Supplementary file 1 [file antibiotics-09-00164-s001.pdf]

Acquisition of Colistin Resistance Links Cell Membrane Thickness Alteration with a Point Mutation in the *lpxD* Gene in *Acinetobacter baumannii*

Neveen M Saleh [1,*], Marwa S Hesham [1], Magdy A Amin [2] and Reham Samir Mohamed [2]

[1] Department of Microbiology, Division of Basic Medical Science, National Organization for Drug Control and Research (NODCAR), 12553 Giza, Egypt; no_more_tears1986@hotmail.com

[2] Department of Microbiology and Immunology, Faculty of Pharmacy, University of Cairo, 11562 Cairo, Egypt; magdy.amin@pharma.cu.edu.eg (M.A.A.) reham.samer@pharma.cu.edu.eg (R.S.)

* Correspondence: salehneveen@yahoo.com; neveensaleh0@gmail.com; Tel.:02-01006758687

Table S1: Comparison of Colistin MIC determination using disc diffusion and microdilution method for selected clinical A. baumannii isolates

A.baumannii **strain no.**	**Colistin-MIC[1] microdilution**	**Colistin susceptibility disc diffusion**
**MS7d**	<0.125	R
**MS32d**	512	R
**MS34d**	>512	R
**MS48d**	0.25	R
**MS1**	64	R
**MS18**	64	R
**MS30**	<0.125	R
**MS37d**	0.25	R
**MS47d**	512	R
**MS50**	<0.125	S
**MS64**	<0.125	S

[1] The CLSI has selected a MIC of ≥2 μg/ml as susceptible and a MIC of ≥4 μg/ml as resistant to colistin (CLSI).
